# Nuclear track autoradiography for radon-related radiobiological hazards in Abu-Sannan Petroleum Area, Egypt

**DOI:** 10.1038/s41598-025-23659-8

**Published:** 2025-11-12

**Authors:** Aya Abdelrazk, Hagar Ahmed, Ali El-Farrash, Mohamed Mitwalli

**Affiliations:** 1 Nuclear, Radiation and High Energy Physics Laboratory, Physics Department, Faculty of Science, Mansoura University, P.O.B. 35516, Mansoura, Egypt; 2 Interdisciplinary Research Center for Industrial Nuclear Energy (IRC-INE), King Fahd University of Petroleum and Minerals (KFUPM), P.O.B. 31261, Dhahran, Saudi Arabia

**Keywords:** Petroleum, Radioactivity, Radiobiological impacts, Radiation dose, Nuclear detector, Environmental sciences, Natural hazards, Solid Earth sciences

## Abstract

Abu-Sannan Petroleum Area (ASPA) is situated in Egyptian Western Desert and presents a suitable site for oil exploration. This study aimed to evaluate the radiological hazards linked to technologically enhanced naturally occurring radioactive materials (TENORM), particularly Radium (²²⁶Ra) and Radon (²²²Rn), in the General Petroleum Y-Field (GPY) situated in the northeastern area of ASPA. The study was examined significant deficiencies in radiological monitoring by evaluating ²²²Rn dynamics, geological correlations, and related radiation hazards. CR-39 nuclear track detector was used to examine 23 sedimentary samples for ²²²Rn and ²²⁶Ra radioactivity concentration. The samples under study were taken from depths 100 to 2600 m ASPA. A radioecological assessment of shale, limestone, and sandstone formations was also conducted. Results showed that the radioactivity concentrations of ²²²Rn and ²²⁶Ra increased with depth, reaching a maximum of 1906.85 ± 194.52 Bq.m^−3^ and 89.99 ± 1.69 Bq.m^−3^ at 2600 m, respectively. The value of ²²²Rn and ²²⁶Ra were higher than the limits set by the IAEA and UNSCEAR. The annual effective doses (AED) varied from 7.41 ± 4.82 to 48.11 ± 12.27 m.Sv.y^− 1^, exceeding the international safety threshold of 1–10 m.Sv.y^− 1^. Increased hazards were observed in deeper layers, notably the Bahariya Sandstone, due to the presence of uranium-rich rocks and highly porous matrices. Radiometric and geological analyses indicated that shale strata contain ²²⁶Ra but cracked limestone and sandstone formations permit the migration of ²²²Rn. Principal component analysis (PCA) revealed significant correlations among ²²²Rn concentration, exhalation rates, and AED, underscoring the lithological influence on radiation hazards. The results highlight considerable occupational health hazards in ASPA, necessitating improved radiation protection measures and ongoing surveillance to reduce lung cancer risks associated with prolonged ²²²Rn exposure, in accordance with WHO guidelines. This study offers essential data to inform regulatory strategies in petroleum-producing areas with similar geological characteristics and radiation protection protocols.

**Supplementary Information:**

The online version contains supplementary material available at 10.1038/s41598-025-23659-8.

## Introduction

 Abu-Sannan Petroleum Area (ASPA) is situated between latitudes 29º37’–29º38’ N and longitudes 28º42’–28º43’ E in Egypt’s Western Desert. ASPA is a region characterized by substantial hydrocarbon and oil extraction operations. Petroleum development and production have increased the presence of technologically enhanced naturally occurring radioactive materials (TENORM), which often mobilize primordial elements such as Uranium (²³⁸U) and Thorium (²³²Th). The extraction of TENORM from deep geological formations to the surface results in increased levels of natural radioactivity^1–4^. ²³⁸U and ²³²Th decay into Radon (²²²Rn), which a radioactive gas and classified as primary carcinogen factor by the International Agency for Research on Cancer (IARC)^5^ and world health organization (WHO)^6^. ²²²Rn geological properties, including its migration through clay, slit, sandy gravel, porous soils as well as fractured bedrock, make it a critical harmful pollutant in oil and gas fields, where naturally occurring radioactive materials NORM and TENORM accumulate in drilling equipment, pipelines, and produced water^7–10^. The production of oil and gas in ASPA involves sedimentary basins rich in organic matter and uranium-bearing minerals, which act as accumulation hazards of radionuclides, particularly ²²²Rn isotopes^11^. During extraction, ²²²Rn gas is released into the environment, posing radiological impacts to occupational and public. Chronic exposure to ²²²Rn and its progeny (²¹⁸Po, ²¹⁴Pb, ²¹⁴Bi) increases the risk of lung cancer due to alpha particle emission during decay^3,6,12^. Furthermore, ²²²Rn interaction with geological processes associated with hydrothermal, soil permeability, lithology, and groundwater flow control its distribution pattern which necessitates radiation protection studies^13^.

To quantify ²²²Rn radioactivity concentration levels, CR-39 solid state nuclear track detectors (SSNTD) are widely employed due to their high sensitivity, cost-effectiveness, and ability to integrate measurements over extended period^14–17^. Nuclear polymeric CR-39 detectors is an auto-record for alpha particle tracks emissions from ²²²Rn decay. In ASPA, where petroleum activities particularly northeastern area of ASPA, understanding ²²²Rn dynamics is essential to mitigating environmental radioactivity, safeguarding public health and accurate exposure data critical for risk assessment.

Previous studies have primarily focused on geological formations, groundwater, and construction materials, while limited data exist on radioactivity level in ASPA. By addressing ²²²Rn behavior in the ASPA study provides new insights into radiological impact in one of Egypt’s most significant Egypt’s national outputs of gas and oil exploration. Given the narrow scope of the study in the region which reported ²²²Rn radioactivity concentrations exceeding the WHO recommended level of 100  Bq.m^−3^^6,18^. However, data specific to ASPA remain scarce, despite its unique geological profile characterized by Cretaceous limestone and shale formations^19,20^. These lithological units influence ²²²Rn emanation rates, as fractured limestone enhances gas mobility, while shale acts as a barrier^21^. Biological effects of ²²²Rn are dose-dependent, with prolonged exposure leading to DNA damage and cellular mutations referring IARC^22^. Occupational hazards are particularly pronounced in oilfield workers, who face dual exposure risks from inhalation and dermal contact with TENORM contaminated materials^4,12^. Additionally, ²²²Rn synergy with other harmful pollutants, such as volatile organic compounds (VOCs) in petroleum, may exacerbate respiratory ailments^23^.

Most radiological assessments in Egypt have been limited to a few oilfields, despite the known risks of ²²²Rn in petroleum-producing regions. The geologically distinct ASPA, which is composed of Cretaceous limestone and shale strata, has little to no published information on ²²²Rn levels. Despite being poorly studied from a radiological perspective, these lithological units have a significant impact on ²²²Rn mobility and emission. In addition to, the combined effects of geological formations, petroleum operations, and residential proximity on ²²²Rn buildup and dissemination are often overlooked in current research as reported in EPA and IAEA^3,24,25^. As a result, comprehensive risk assessment models that are specific to ASPA are lacking, particularly those that incorporate statistical tools like discriminant functions and correlation indicators. This study employed CR-39 SSNTD to investigate ²²²Rn radioactivity in ASPA in a systematic manner. The source of the innovation aims to supply basic information on ²²²Rn activity levels in a region rich in petroleum that lacks thorough documentation. Examining the relationship between ²²²Rn dispersion and the geological features (shale versus fractured limestone) that distinguish ASPA. Evaluating the health impacts on nearby communities and occupational workers using dose conversion models. Applying statistical techniques (discriminant analysis and correlation coefficients) to assess ²²²Rn dynamics in petroleum environments and differentiate lithological influences.

## Experimental analysis

### Sampling strategy

**Fig. 1:**
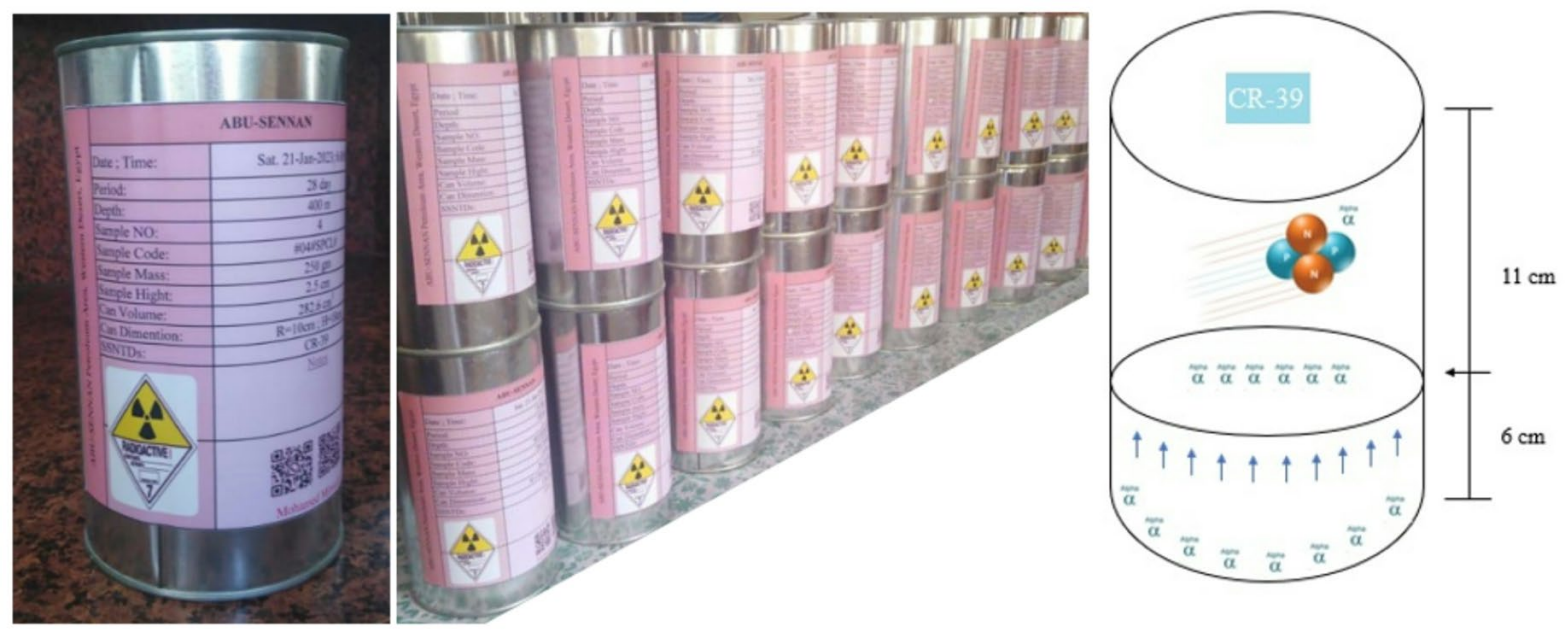
Set-up of nuclear autoradiographic system used to determine ²²²Rn radioactivity concentrations.

Sampling strategy fulfilled technical protocols of internation atomic energy agency (IAEA TECDOC-1415)^3,25^ and Egyptian Geological Survey and Mining Authority. Sampling locations were selected to represent diverse lithological units, including shale, limestone, and sandstone, to account for variations in geological properties influencing ²²²Rn emanation. A total of 23 sedimentary samples were systematically collected from ASPA within different depths ranging between 100 and 2600 m. The samples under study were air-dried, homogenized by grinded to fine powder then sieved < 1 mm to remove organic debris and residuals. Prepared samples were sealed in gas-tight metal containers (1.5 L volume) for 28 days to allow secular equilibrium between ²²⁶Ra and ²²²Rn as shown in Fig. 1. Polymeric nuclear CR-39 detectors (American Technical Plastics, 600 μm thickness) were mounted inside the containers, ensuring direct exposure to emitted ²²²Rn gas as shown in Fig. 1. After 28 days (irradiation period), detectors were retrieved and chemically etched in 6.25 N NaOH at 70 ± 1 °C for 7 h to amplify alpha particle tracks^26,27^. Track densities (ρ = tracks.cm⁻²) were quantified using an optical microscope (400x magnification).

Control and uncertainty of CR-39 detector were analyzed by (^241^Am and ^226^Ra) certified reference materials (CRM) to validate detector efficiency. A gamma spectrometer NaI(Tl) was employed no more than for validation purposes to cross-check ²²⁶Ra radioactivity concentrations obtained from CR-39 SSNTD. The validation process was conducted for energy and efficiency calibration by using certified reference materials (^241^Am and ^226^Ra), following standard protocols of IAEA^4,12^ to ensure reliability. The radioactivity limit of ²²⁶Ra obtained from the NaI(Tl) detector was approximately 15 Bq.kg⁻¹, which is consistent with previously research. Quality control was matched through routine background analysis and repeated measurements, demonstrating uncertainties within ± 10%. The comparative method showed less than 18% deviation from CR-39 outcomes, in agreement with previous experimental studies, thereby confirming the robustness of the radioanalytical methodology of ^222^Rn^17,28,29^.

### Theoretical calculations

For calculating the concentration of ²²²Rn and ^226^Ra at secular equilibrium the following equations (1 and 2) were applied to derive ^222^Rn and ^226^Ra radioactivity concentration in Bq.m^−3^ and its radiological parameters:

where: C_Rn_ is ^222^Rn activity concentration (Bq.m^-3^), ρ is the track density (track.cm^-2^), T is the irradiation time, and η is the factor of detector calibration^30^, where $\alpha$is the total count of tracks,* f* is the matrix fields of investigated, $\pi {r^2}$ is the studied fields area^15,30,31^

1 $${C_{Rn}}=\frac{\rho }{{\eta T}}\; \pm \;\frac{{\sqrt {\frac{\alpha }{{f\pi {r^2}}}} }}{{\eta T}}$$

2 $$\rho =\frac{\alpha }{{f\pi {r^2}}}\; \pm \sqrt {\frac{\alpha }{{f\pi {r^2}}}}$$

For calculating the ^222^Rn surface and mass exhalation rate, the following equations (3-5) were used: where E_A_ is surface exhalation rate (Bq.m.^−2^h.^−1^), E_M_ is mass exhalation rate (Bq.kg.^−1^h.^−1^), λ is the decay constant of ^222^Rn (2.10 × 10 –6 s^− 1^), C_Rn_^222^Rn concentration (Bq.m^−3^), V is the Can volume (m^3^), A is the surface area of sample (m^2^), M is the sample mass (kg), T is the irradiation time^30,32^, and T_e_ is effective time related to the actual irradiation time of ^222^Rn^30,33,34^.

3 $$E_{A} = \frac{{C_{{Rn\,}} \;V\,\lambda }}{{A\;T_{e} }} \pm \frac{{\sqrt {\frac{\alpha }{{f\pi r^{2} }}} \;V\,\lambda }}{{\eta TA\;T_{e} }}$$

4 $$E_{M} = \frac{{C_{{Rn\,}} \;V\,\lambda }}{{M\;T_{e} }} \pm \frac{{\sqrt {\frac{\alpha }{{f\pi r^{2} }}} \;V\,\lambda }}{{\eta TM\,T_{e} }}$$

5 $$T_{e} \; = [T + \frac{1}{\lambda }(e^{{ - \lambda T}} - 1)]$$

The following equation (6) was used to estimate the annual effective dose $AED$: where D_E_ is the annual effective dose, A is the conversion coefficient, H is the indoor occupancy factor, F is the indoor equilibrium factor between ²²²Rn and its progeny, and T is the indoor exposure time in hours per year^35,36^.

6 $${D_E}=\,A\,H\,F\,T\,{C_{Rn}}\;$$

The working levels were calculated by using the following equation (7): where ${C_{Rn}}$ is ²²²Rn concentration in Bq.m^-3^,* f* is the equilibrium factor for ²²²Rn, ICRP, ^35,37–39^,

7 $$WL=\frac{{{C_{Rn}}\;x\;f}}{{3700}}\; \pm \;\frac{{\sqrt {\frac{\alpha }{{f\pi {r^2}}}} \;x\;f}}{{\eta T\;x\;3700}}$$

The specific activity ^226^Ra (Bq.kg^-1^) was calculated by the following equation (8): where *C*_Ra_ is the effective ^226^Ra content of investigated samples (Bq.kg^-1^), *M* is the mass of sample (kg), *A* is the area of cross-section of the container, *h* is the distance between the detector and the top of the fixed station, *f* is the sensitivity factor and *T*_*e*_ is the effective exposure time^16,26^.

8 $${C_{Ra}}\;=\frac{{\alpha hA}}{{Mf\pi {r^2}\;{T_e}}} \pm \frac{{hA\;\sqrt {\frac{\alpha }{{f\pi {r^2}}}} }}{{M\;{T_e}}}$$

^222^Rn radiation index (Alpha index) originating from the exploration and geological process was calculated by the following equation (9): where Iα is the alpha radiation index, *C*_Ra_ is the ^226^Ra content of investigated samples (Bq.kg^-1^), and 200 is the conversion coefficient^26,40^.

9 $$I\alpha =\frac{{{C_{Ra}}}}{{200}}$$

### Calibration and limitation

To ensure the accuracy of the measurements, calibration data for the CR-39 detector were considered as detailed in the supplementary material. The detector’s calibration factor (K), which relates track density to ²²²Rn concentration, was determined elswhere previous experimental studies^28^. The minimal detectable limit (MDL) and measurement uncertainty were assessed to determine the dependability of the data. The sensitivity of the CR-39 NTD to ²²²Rn was determined to be 0.223 tracks/cm² per Bq.m^−3^. The Minimum Detectable Limit (MDL) and Measurement Uncertainty for the CR-39 detector were computed using the supplied calibration data. The MDL is the minimal ²²²Rn exposure that can be accurately differentiated from the lowest measured level, as determined by equations (10 and 11):

10 $$MDL=\frac{{3\;{\sigma _{blank}}}}{k}$$

wher the standard deviation of blank readings (track density without ²²²Rn exposure) is denoted as k, which represents the calibration factor of CR-39 NTD.

The overall uncertainty in ²²²Rn exposure (E) is obtained from the uncertainty in track density measurement (σT = 10.97%⋅T) and the uncertainty of the calibration factor, which is determined by weighted regression variance using error propagation for E = T/k:

11 $$\frac{{\sigma E}}{E}=\sqrt {{{\left( {\frac{{\sigma T}}{T}} \right)}^2}+{{\left( {\frac{{\sigma k}}{k}} \right)}^2}}$$

The minimum detectable limit for ²²²Rn concentration was determined to be 121.07 Bq.m^−3^, guaranteeing testing accuracy even at low ²²²Rn levels. The overall uncertainty in ²²²Rn measurements was 10.97%, consistent with IAEA norms^4,12,25,41^.

## Results and discussion

### Depth profiles of radon and radium

**Fig. 2:**
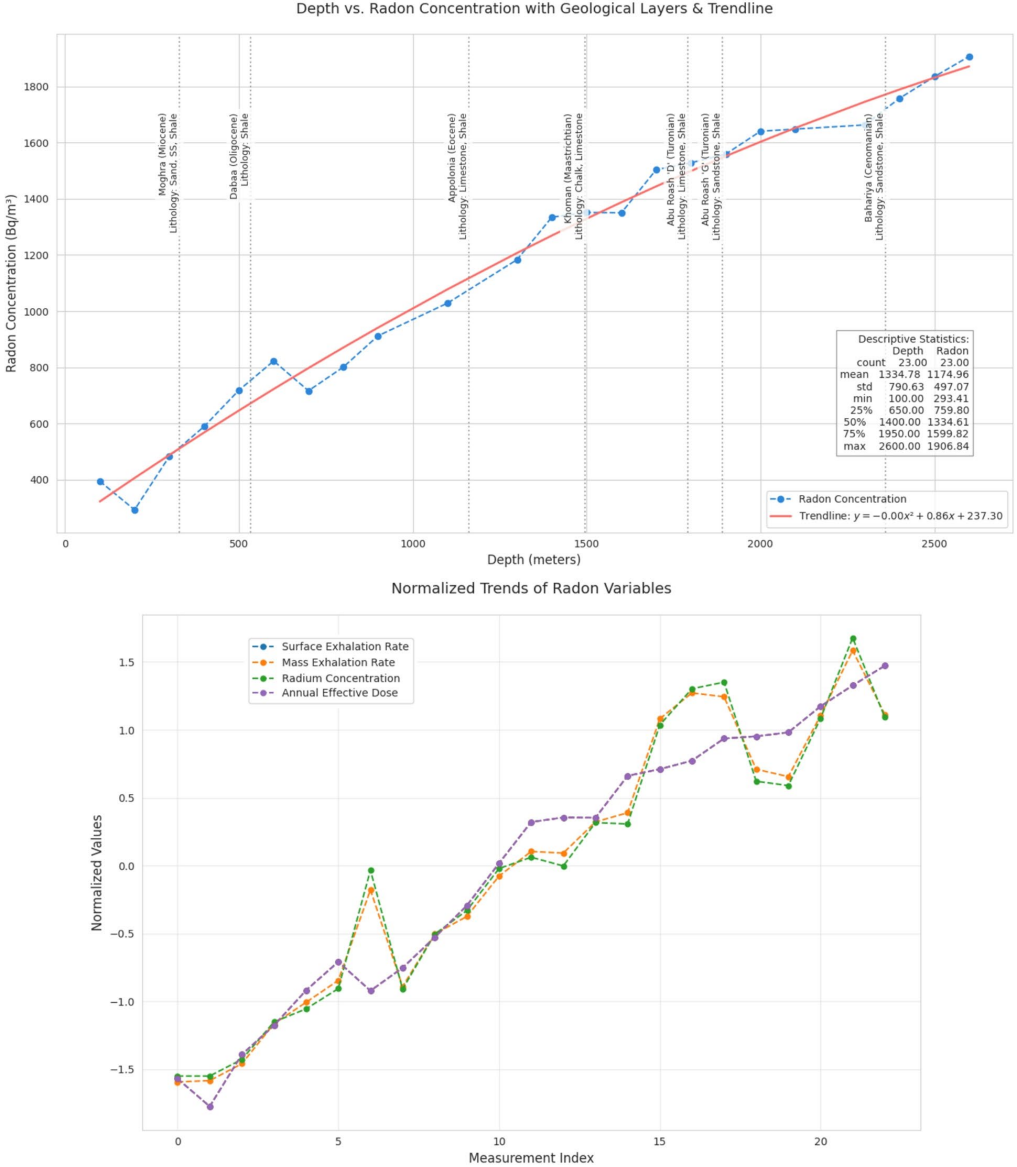
Overall comparsion of ²²²Rn concentration and other radiation paramter: ²²²Rn concentration generally increases with depth, except for minor dips at 200 m (293 Bq.m^−3^) and 700 m (717 Bq.m^−3^). Irregularities: A temporary plateau occurs between 1400–1600 m (concentration stabilizes around ~ 1350 Bq.m^−3^). Sharp increases are seen from 2300 m to 2600 m (244 Bq.m^−3^ over 300 m). Geological annotations: (i) Khoman Formation (1495 m): Chalk and limestone correlate with a stabilization in ²²²Rn levels; (ii) Bahariya Formation (2360 m): Sandstone/shale layers align with the steepest ²²²Rn increase.

The radioactivity concentrations of ²²²Rn and ²²⁶Ra at different depths are summarized in Table 1 and illustrated in Fig. 2. Results reveal a progressive increase in ²²²Rn activity with depth, peaking at 2600 m (1906.85 ± 194.52 Bq.m^−3^). This trend reflects increased ²²²Rn emanation from deeper formations, likely associated with higher ^238^U/^226^Ra content and increased porosity within specific lithologies. ²²⁶Ra radioactivity concentrations also exhibit a general upward trend with depth, albeit with fluctuations. Notable spikes occur at 700 m (51.95 ± 1.56 Bq.m^−3^) and 2400 m (76.79 ± 1.47 Bq.m^−3^), aligning with shale–sandstone sequences known to retain ^226^Ra. An anomaly at 2100 m (66.52 Bq.m^−3^) may indicate either a lithological transition reduced shale content or measurement variability.

These results of ^222^Rn and ^226^Ra were exceeded recommended limits by ICRP^42^ and UNSCEAR^35,37^ in the advanced case regarding exploration process and accumulation of TENORM in work Y-Field, GPY of ASPA. This is particularly important in lithologies with high silica content, where ^238^U tends to be more concentrated^43^. Such an increase is consistent with deeper strata often containing ^238^U and ^232^Th bearing minerals, such as zircon, monazite, and uraninite, embedded within granitic or metamorphic lithologies^44,45^. The decay of ^226^Ra, a ^238^U series radionuclide, is the primary source of ²²²Rn, and higher radioactivity concentrations at depth may be linked to less weathered mineral phases and reduced ²²²Rn loss to the atmosphere. In fractured granitic lithologies, ²²²Rn migration is enhanced by microfractures and joints, which can act as conduits for gas transport^46^. Conversely, in compact, low-permeability sedimentary rocks such as shales, ²²²Rn mobility is reduced, although emanation may still be high if equivalent ^238^U content is significant. Notable increase of ^226^Ra radioactivity concentrations indicate that lithologies at greater depths have higher content radionuclides from ^238^U decay series. In ^238^U rich granitoids and certain metamorphic lithologies (gneisses, schists), ^226^Ra is preferentially retained within mineral lattices such as feldspar and apatite^4,12,47,48^. Anomalies can be attributed to lithological and structural, where variations in elemental composition and porosity affect radionuclide accumulation as well as ²²²Rn emanation. For different depth range, intervals correspond to shear zones affected by hydrothermal alteration and related structural features, which improve fluid migration and mobility of ^238^U. in addition to geological processes can accelerate enrichment of ^238^U content and its decay products, thereby explaining the radioactivity concentrations at depth. The result is consistent with previous observations of ^238^U distribution pattern in fractured and hydrothermally altered sedimentary environments.

**Table 1:** ²²²Rn and ^226^Ra radioactivity concentration, surface and mass exhalation rates of the investigated samples.

No.	Depth	²²²Rn	E_A_	E_M_	^226^Ra
	m	Bq.m^−3^	Bq.m.^−2^h^−1^	Bq.k.^−1^h^−1^	Bq.m^−3^
1	100	394.9 ± 88.52	0.62 ± 0.14	0.02 ± 0.01	18.27 ± 0.74
2	200	293.41 ± 76.3	0.46 ± 0.12	0.02 ± 0.01	18.27 ± 0.86
3	300	483.49 ± 97.95	0.75 ± 0.16	0.03 ± 0.01	21 ± 0.77
4	400	589.75 ± 108.18	0.92 ± 0.17	0.03 ± 0.01	27.12 ± 0.9
5	500	718.7 ± 119.42	1.12 ± 0.19	0.04 ± 0.01	29.27 ± 0.88
6	600	822.98 ± 127.79	1.28 ± 0.2	0.04 ± 0.01	32.58 ± 0.92
7	700	716.89 ± 119.27	1.11 ± 0.19	0.06 ± 0.01	51.95 ± 1.56
8	800	800.93 ± 126.07	1.24 ± 0.2	0.04 ± 0.01	32.49 ± 0.93
9	900	912.3 ± 134.55	1.41 ± 0.21	0.05 ± 0.01	41.5 ± 1.11
10	1100	1028.89 ± 142.88	1.59 ± 0.23	0.05 ± 0.01	45.37 ± 1.14
11	1300	1183.57 ± 153.25	1.83 ± 0.24	0.06 ± 0.01	52.24 ± 1.22
12	1400	1334.61 ± 162.73	2.07 ± 0.26	0.06 ± 0.01	54.08 ± 1.19
13	1500	1351.38 ± 163.75	2.09 ± 0.26	0.06 ± 0.01	52.66 ± 1.15
14	1600	1350.39 ± 163.69	2.09 ± 0.26	0.07 ± 0.01	59.75 ± 1.31
15	1700	1503.7 ± 172.73	2.33 ± 0.27	0.07 ± 0.01	59.53 ± 1.24
16	1800	1528.17 ± 174.13	2.37 ± 0.27	0.09 ± 0.01	75.72 ± 1.56
17	1900	1559.17 ± 175.89	2.41 ± 0.28	0.09 ± 0.01	81.67 ± 1.66
18	2000	1640.47 ± 180.42	2.54 ± 0.28	0.09 ± 0.01	82.74 ± 1.64
19	2100	1648.18 ± 180.84	2.55 ± 0.28	0.08 ± 0.01	66.52 ± 1.32
20	2300	1662.91 ± 181.65	2.57 ± 0.29	0.08 ± 0.01	65.81 ± 1.3
21	2400	1758.02 ± 186.77	2.72 ± 0.29	0.09 ± 0.01	76.79 ± 1.47
22	2500	1834.61 ± 190.8	2.84 ± 0.3	0.1 ± 0.01	89.99 ± 1.69
23	2600	1906.85 ± 194.52	2.95 ± 0.31	0.09 ± 0.01	76.99 ± 1.42

**Fig. 3:**
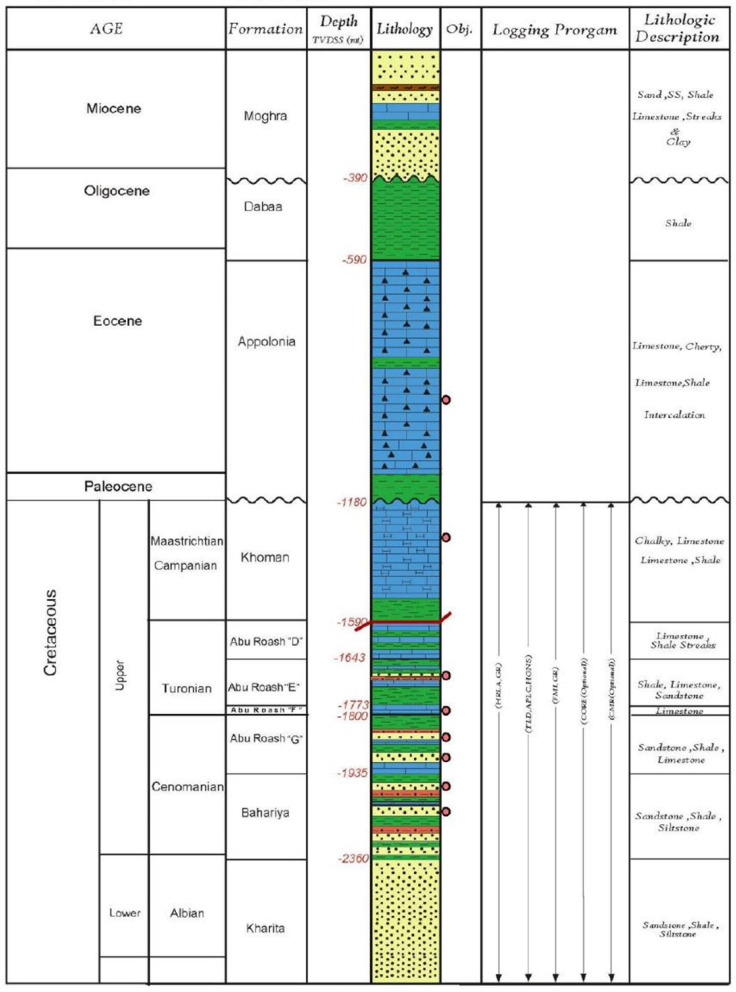
Petroleum stratigraphic column represents formation names, geologic ages, depths, lithologies, and lithologic descriptions of General Petroleum Y-filed (GPY), northeast of ASPA.

### Exhalation rates and radiological parameters

Both of the surface (E_A_) and the mass exhalation rates (E_M_) increase with depth, consistent with the trends of ²²²Rn and ^226^Ra presented in Table 1. Maximum values of 2.84 ± 0.3 Bq.m^−2^.h^−1^ (E_M_), and 0.1 ± 0.01 Bq.kg.h^−1^ (E_A_) are recorded at 2500 m within sandstone-rich intervals. The annual effective dose (AED) ranges from 7.41 ± 4.82 mSv.y^−1^ at 100 m to 48.11 ± 12.27 mSv.y^−1^ at 2600 m. These values exceed the WHO recommended limits of 1–10 mSv.y^−1^^6^, indicating significant occupational and environmental exposure risks in deeper horizons referring to ICRP^42^ and UNSCEAR^35,37^. Based on the progressive increase in ^222^Rn and ^226^Ra with depth, coupled with elevated E_A_ and E_M_, it is likely that deeper intervals intersect ^238^U rich lithologies, possibly granitoid intrusions or metamorphic basement rocks. The peaks at 700 m, 1800 m, and 2500 m may correspond to lithological transitions, such as faulted zones or contact zones between sedimentary cover and crystalline basement, where ²²²Rn mobility is enhanced due to fracturing^49^.

**Table 2:** The elemental composition of each lithology depends largely on the mineralogy and depositional environment.

Lithology	Major Elements Likely Present	Radioactivity Implications	References
Sandstone	Si, Al, K, Fe (quartz + feldspars + clays)	Potassium from feldspar/mica can raise ⁴⁰K levels.	^50,51^
Shale	Si, Al, K, Fe, trace U & Th in clays and organic matter	Clays are a main source of ⁴⁰K and adsorbed ^238^U, ^232^Th.	^52,53^
Limestone	Ca, Mg (calcite, dolomite), minor Si, Al	Low radioactivity unless it contains phosphates or organic matter.	^54,55^
Chert	Si (microcrystalline quartz)	Low intrinsic radioactivity, except for impurities.	^56^
Sand/Shale mix	Combination of above	Radioactivity depends on clay and feldspar content.	^52,57^

### Lithological setting on radionuclide distribution

The stratigraphic column illustrates lithological Fig. 3 variations from Miocene to Lower Cretaceous formations, with clear implications for both elemental composition and natural radioactivity profiles. Shale-dominated intervals, such as the Dabaa Formation and clay-rich members of Abu Roash, are expected to exhibit elevated natural gamma readings due to higher concentrations of potassium (⁴⁰K) in illite/mica and potential ^238^U and ^232^Tth adsorption onto clay minerals^52,53,58^ as presented in Table 2. In contrast, pure carbonate intervals (e.g., Khoman, Apollonia) generally display low radioactivity, reflecting their dominance of Ca and Mg carbonate minerals with minimal radioactive element content^54,55^. Sandstone-dominated formations, such as Moghra, Bahariya, and Kharita, show moderate radioactivity dependent on clay matrix proportion and feldspar content^50,51,57^. Depth trends indicate that while lithology remains the primary control, deeper organic-rich shales may display localized ^238^U enrichment under reducing conditions^59,60^. This relationship between lithology, elemental composition, and radioactivity is critical for interpreting radionuclides logs, identifying reservoir-quality intervals, and assessing potential NORM/TENORM related environmental risks during petroleum exploitation as shown in Table 3 as well as petroleum stratigraphic column Fig. 3.

**Table 3:** Correlation with geological formations and radioactivity.

Formation	Depth (m)	Sample No.	Key Observations
Moghra (Miocene)	329	~ 300 m (No. 3)	Sand, SS, Shale: Moderate ²²²Rn (483 Bq.m^−3^) and ^226^Ra (21 Bq.m^−3^). Shale may trap ²²²Rn, limiting exhalation.
Dabaa (Oligocene)	534	~ 500 m (No. 5)	Shale-dominated: Rising ²²²Rn (718 Bq.m^−3^) and ^226^Ra (29 Bq.m^−3^). Low permeability of shale restricts ²²²Rn release, increasing retention.
Apollonia (Eocene)	1161	~ 1100 m (No. 10)	Limestone/Shale: ²²²Rn surges to (1029 Bq.m^−3^). Fractured limestone may enhance ²²²Rn migration, while shale contributes to ^226^Ra retention (45 Bq.m^−3^).
Khoman (Maastrichtian)	1495	~ 1500 m (No. 13)	Chalk/Limestone: Peak ^222^Rn (1351 Bq.m^−3^) and ^226^Ra (53 Bq.m^−3^). Chalk’s porosity facilitates ^222^Rn exhalation, while limestone retains ^226^Ra.
Abu Roash ‘D’ (Turonian)	1792	~ 1800 m (No. 16)	Sandstone/Shale: High ²²²Rn (1528 Bq.m^−3^) and ^226^Ra (76 Bq.m^−3^). Sandstone porosity promotes ^222^Rn release; shale enriches ^226^Ra.
Bahariya (Cenomanian)	2360	~ 2400 m (No. 21)	Sandstone/Shale: Maximum ²²²Rn (1758 Bq.m^−3^) and ^226^Ra (77 Bq.m^− 3^). Porous sandstone enhances ^222^Rn emanation; ^238^U rich shale elevates ^226^Ra.

### Statistical analysis

**Fig. 4:**
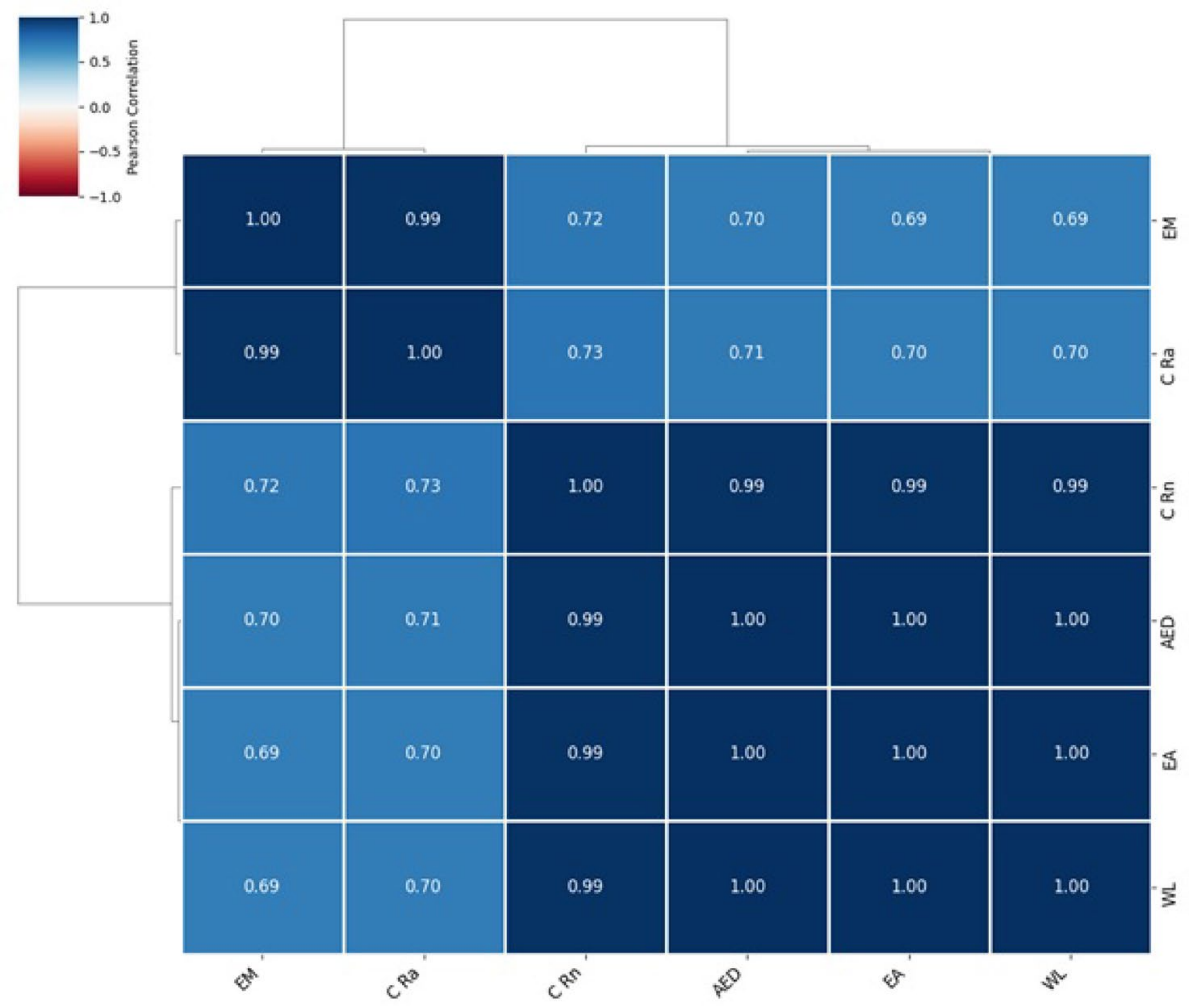
Pearson correlation heatmap with dendrogram for calculated radiological parameters.

**Fig. 5:**
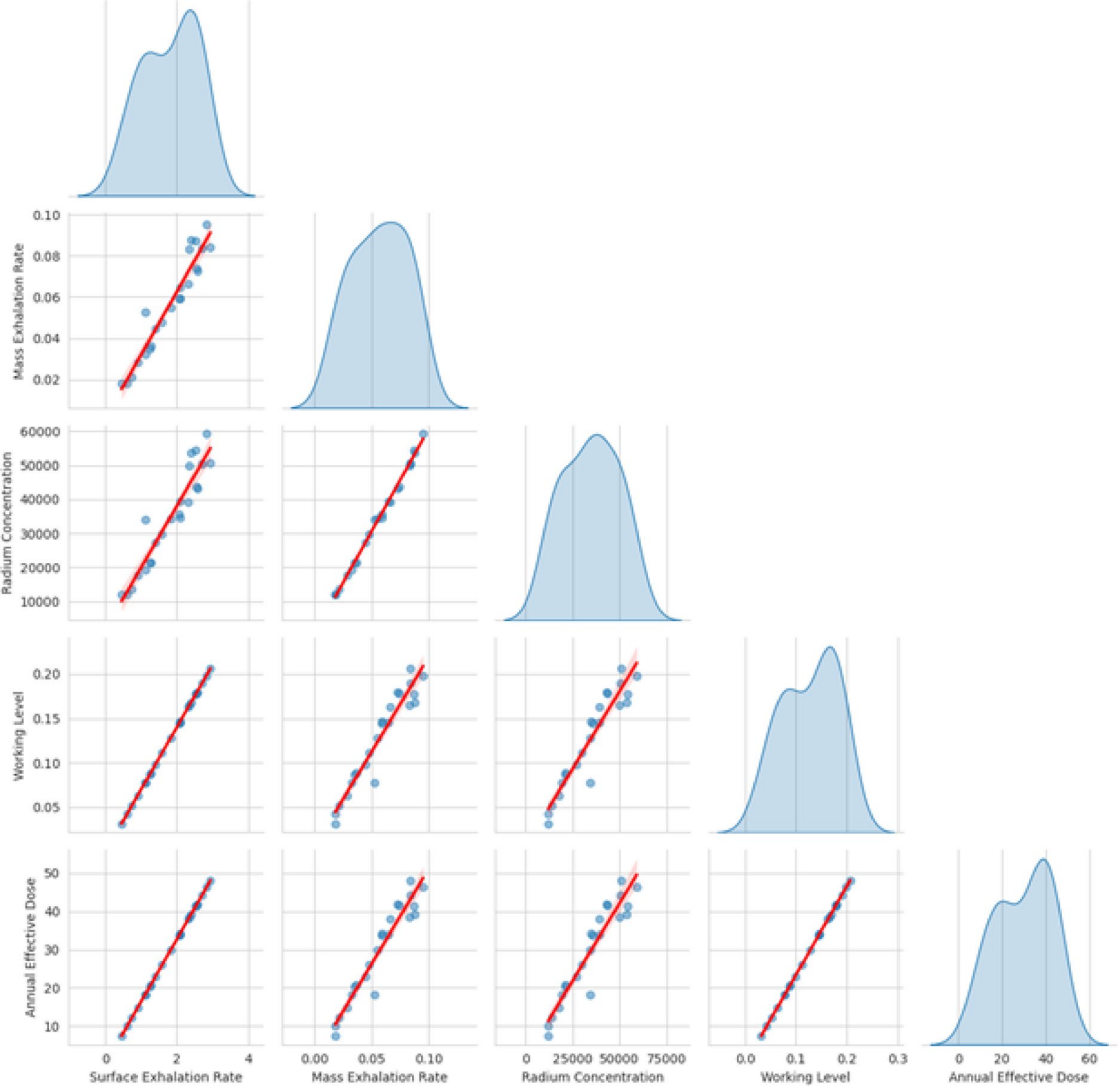
Scatter plot matrix with regression lines; ^226^Ra Concentration shows the most extreme fluctuations (e.g., sudden spikes); Working Level and Indoor Annual Effective Dose follow similar upward trajectories; Surface Exhalation Rate ↔ Indoor Annual Effective Dose: Near-perfect correlation (r^2^ = 0.99); Mass Exhalation Rate ↔ ^226^Ra Concentration: Strong correlation (r^2^ = 0.98).

**Fig. 6:**
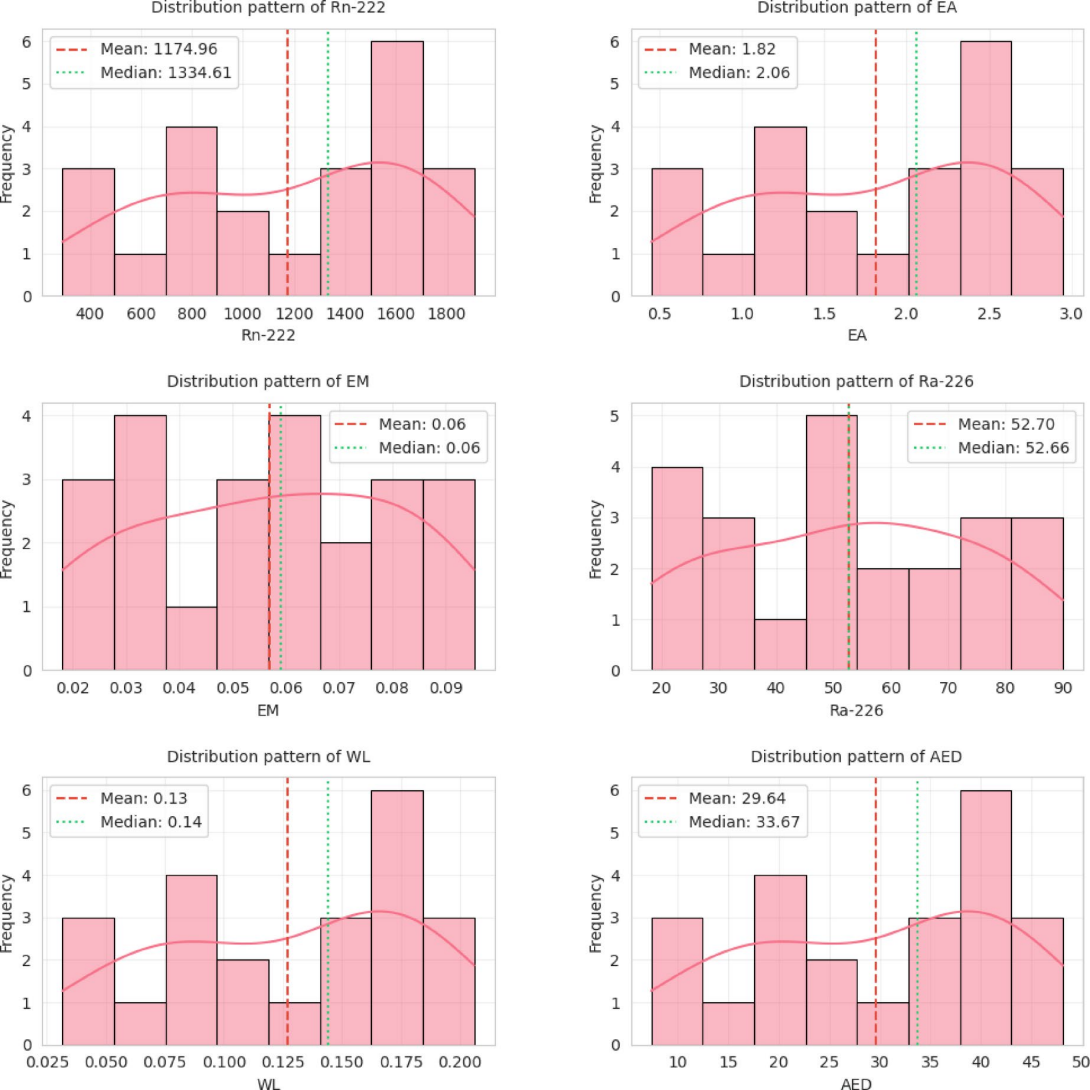
Histograms show actual distribution frequency of the following: (1)^222^Rn: Shows right-skewed distribution with increasing frequency at higher radioactivity concentrations. (2) E_A_: Bimodal distribution suggesting multiple populations. (3) E_M_: Left-skewed distribution with most values < 0.07 Bq.kg^-1^. (4) ^226^Ra: Normal-like distribution with central peak around 50 Bq.kg^-1^. (5) WL: Uniform distribution between 0.03 and 0.20. (6) AED: Right-skewed distribution reflecting radiation exposure risks.

**Fig. 7:**
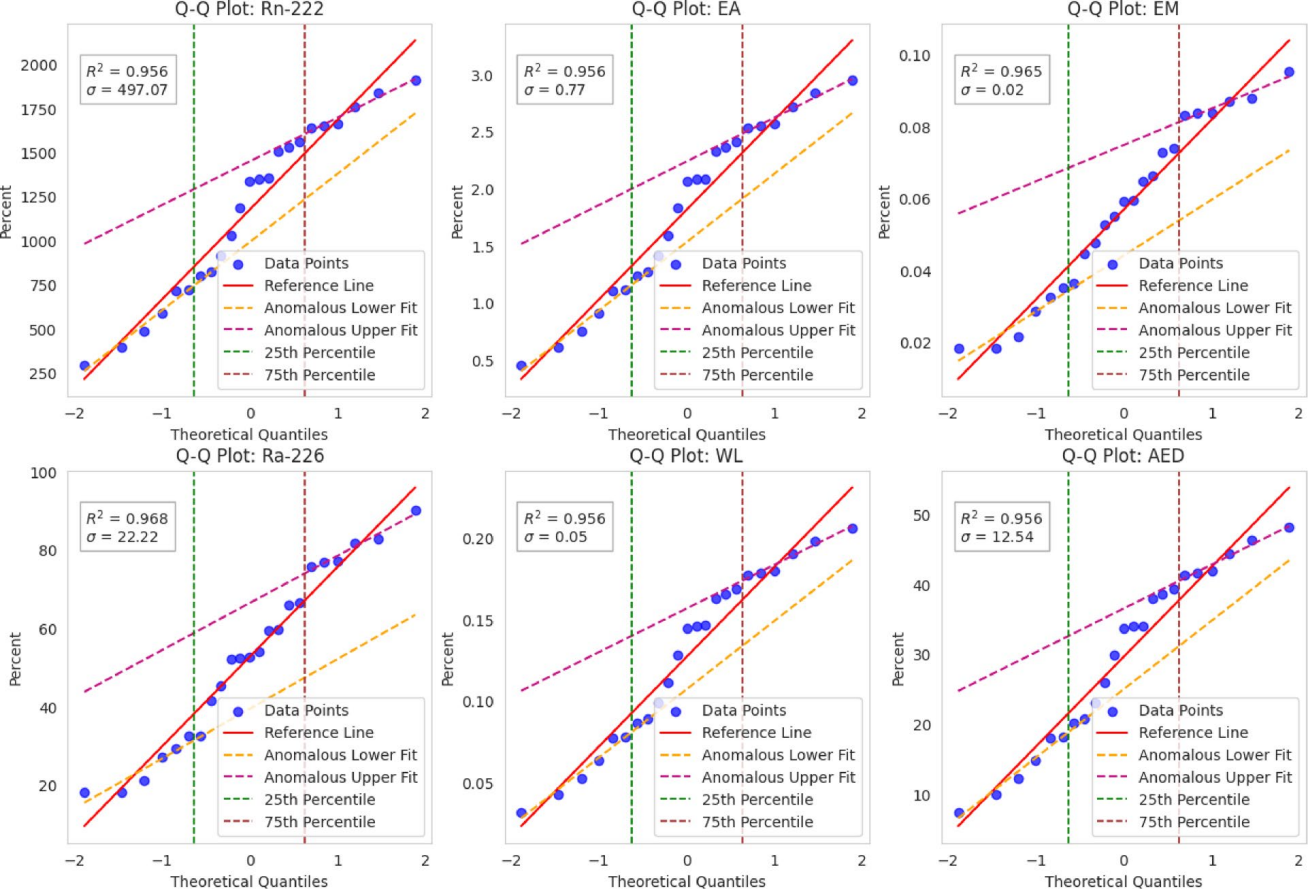
Q-Q plot demonstrated how the data compares to normal distribution.

**Fig. 8:**
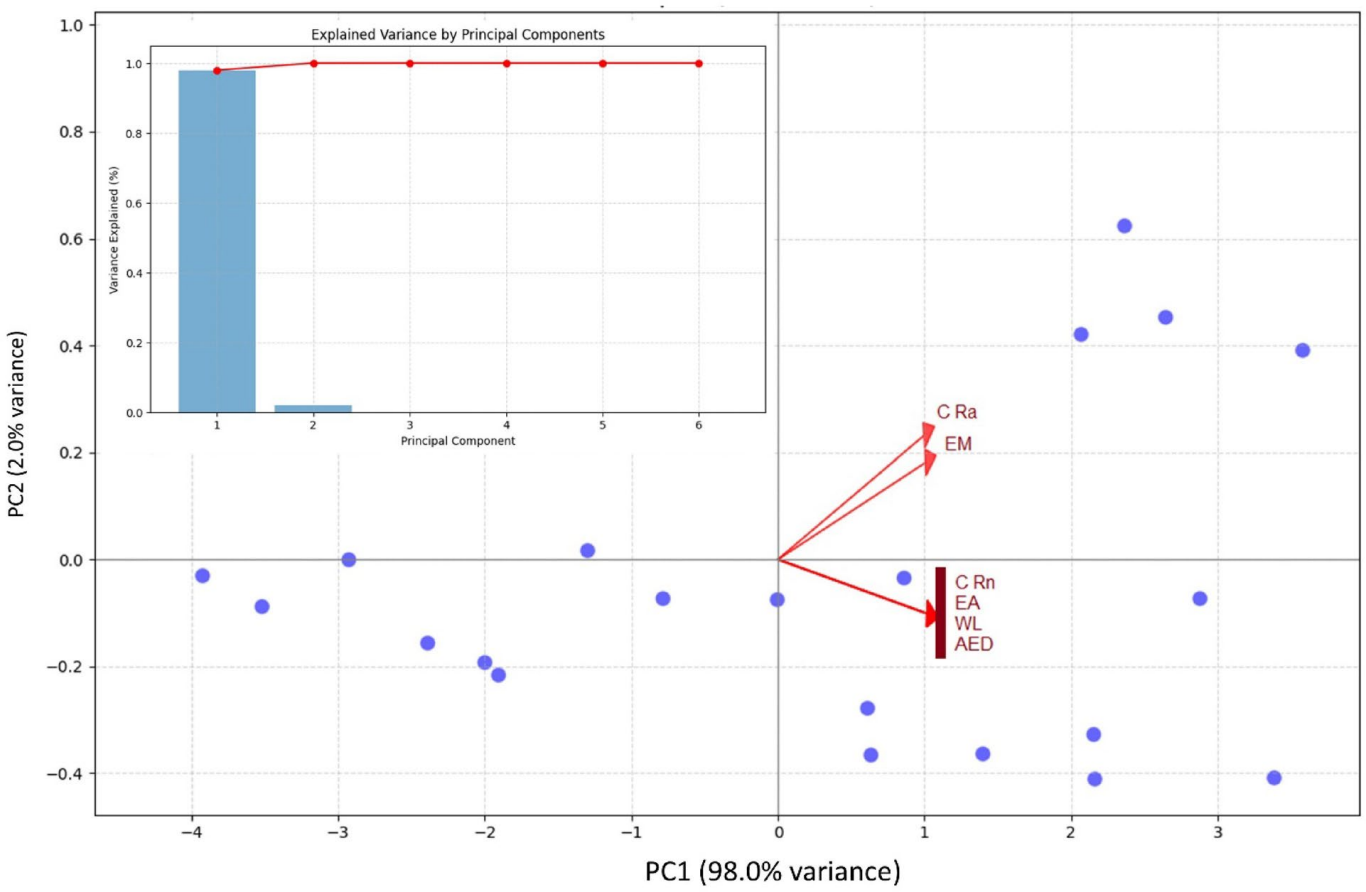
Principal components (PC1 and PC2) explain > 95% of the total variance (PC1 ≈ 90%, PC2 ≈ 5–6%), (PC) means that ^222^Rn, E_A_, E_M_, ^226^Ra, WL, and AED can be effectively visualized in 2D without significant loss of information.

Python and IBM SPSS (V 2.0) statistical tools were employed for all statistical studies of nuclear data interpretation, isotopes correlation and including infographics. Descriptive statistics of the dataset are provided in Table 4, while Fig. 4 shows a Pearson correlation heatmap with dendrogram clustering^61^. Results confirm strong correlations between ^222^Rn activity, and all radiological parameters with overall r^2^ = 0.99 indicated in correlation and frequency plots as shown in Figs. 5 and 6. The normal distribution of the data was evaluated using Quantile–Quantile (Q–Q) plots^62^ and to precisely distinguish between the sample depths. The entire dataset was analyzed with a confidence level of 95% and a significance threshold presented in aligned data of infographics. Q–Q plot in Fig. 7 indicates that the dataset follows a near-normal distribution, validating subsequent statistical inferences. Principal component analysis (PCA) further highlights two dominant variance clusters: PC1 (exposure index): Dominated by ^222^Rn concentration, surface exhalation rate, working level, and AED, accounting for the majority of variance. PC2 (material properties): Governed mainly by mass exhalation rate and ^226^Ra concentration, forming a distinct correlation pattern. Together, PC1 and PC2 explain over 95% of the variance, as shown in Fig. 8.

### Mineralogical and lithological implications

The observed depth-related increase in ²²²Rn and ²²⁶Ra, combined with elevated E_A_ and AED values, suggests deeper intervals intersect ^238^U rich lithologies. Accessory minerals (zircon, monazite, allanite) within sandstones and granitoid intrusions. Fractured crystalline basement rocks, which act as conduits for ²²²Rn migration. Clay-rich intervals, where adsorption and retention enhance ^226^Ra content. By contrast, carbonates and quartz-rich lithologies display lower ²²²Rn emanation unless ^238^U inclusions are present^49,63^.

The KDE curves (red smooth lines) help identify underlying probability distributions. The mean-median relationships indicate skewness direction (mean > median = right skew).

**Table 4:** Descriptive statistics of the samples investigated from ASPA.

Parameters	^222^Rn	EA	EM	^226^Ra	WL	AED
Mean	1174.96	1.82	0.059	31981.18	0.124	29.11
Median	1334.61	2.09	0.064	34648.4	0.146	34.09
S. D.	544.12	0.84	0.023	13233.5	0.056	13.02
Variance	296,065.97	0.7	0.0005	175,176,000	0.0031	169.5
Range	1613.44	2.34	0.077	47,193.89	0.163	40.15
Min	293.41	0.45	0.018	12,016.41	0.032	7.4
Max	1906.84	2.95	0.095	59,209.90	0.206	48.11
Sum	27,024.15	41.79	1.36	735,567.09	2.86	669.59
SEM	113.43	0.18	0.005	2,759.87	0.012	2.71

### Health and environmental implications

The evaluation of alpha index (α), working level (WL), and annual effective dose (AED) at various depths offers essential insights into the health and environmental hazards linked to ²²²Rn exposure in the ASPA. The α-index is a commonly utilized radiological metric for assessing the ²²²Rn -emitting capability of construction and industrial materials. ICRP^38,39,64^ and UNSCEAR^35,37^ recommend that α values over 0.3 signify materials with possible radiation risks. In ASPA, α progressively increases with depth, ascending from 0.09 at 100–200 m to 0.45 at 2500 m, with multiple depths (1600–2000 m, 2400–2600 m) nearing or beyond the 0.3 threshold. The increased readings indicate substantial lithological enrichment of ^238^U decay radionuclides at deeper levels, which could pose TENORM-related concerns if these formations are disrupted during drilling or extraction. The working level (WL) is an occupational exposure metric that measures the concentration of ²²²Rn decay products in the air. The WL values in ASPA vary from 0.09 ± 0.04 at 200 m to 0.22 ± 0.09 Bq.m^−3^ at 2600 m, indicating a continuous increase with depth. While the majority of WL values are below the globally accepted occupational limit of 0.3 WL^6,35,37^, the higher levels are nearing the crucial threshold. Prolonged occupational exposure in inadequately ventilated underground or drilling settings may increase effective dose loads, particularly for petroleum workers who remain for extended periods in high-²²²Rn areas. AED quantifies health risk by indicating the effective radiation dose received by individuals from breathed ²²²Rn and its decay products. AED values in ASPA exhibit a significant rise with depth, rising from 7.41 ± 4.82 m.Sv.y^− 1^ at 200 m to 48.11 ± 12.27 m.Sv.y^− 1^ at 2600 m.

These results significantly beyond the WHO-recommended yearly exposure range of 1–10 m.Sv.y^− 1^^6^. Depth intervals over 1400 m consistently exceed 30 m.Sv.y^− 1^, presenting substantial health hazards to both occupational workers and adjacent people if ²²²Rn is released into the surrounding environment. The robust link between ²²²Rn /^226^Ra activity and AED indicates that lithological factors practically shale and ^238^U rich sandstone significantly influence ²²²Rn concentration and migration (Table 5).

**Table 5:** Annual effective dose, work level and radiological impact of alpha particle for the investigated samples.

No.	Depth	α	WL	AED
	m	Index	Bq.m^−3^	m.Sv.y^− 1^
1	100	0.09	0.1 ± 0.05	9.97 ± 5.59
2	200	0.09	0.09 ± 0.04	7.41 ± 4.82
3	300	0.1	0.11 ± 0.06	12.2 ± 6.18
4	400	0.14	0.12 ± 0.07	14.88 ± 6.83
5	500	0.15	0.14 ± 0.08	18.14 ± 7.54
6	600	0.16	0.14 ± 0.09	20.77 ± 8.06
7	700	0.26	0.14 ± 0.08	18.09 ± 7.53
8	800	0.16	0.14 ± 0.09	20.21 ± 7.96
9	900	0.21	0.15 ± 0.1	23.02 ± 8.49
10	1100	0.23	0.16 ± 0.1	25.96 ± 9.02
11	1300	0.26	0.17 ± 0.09	29.86 ± 9.67
12	1400	0.27	0.18 ± 0.06	33.68 ± 10.27
13	1500	0.26	0.18 ± 0.1	34.1 ± 10.33
14	1600	0.3	0.18 ± 0.09	34.07 ± 10.33
15	1700	0.3	0.19 ± 0.07	37.94 ± 10.9
16	1800	0.38	0.2 ± 0.09	38.56 ± 10.99
17	1900	0.41	0.2 ± 0.1	39.34 ± 11.1
18	2000	0.41	0.2 ± 0.09	41.39 ± 11.38
19	2100	0.33	0.2 ± 0.08	41.59 ± 11.41
20	2300	0.33	0.2 ± 0.08	41.96 ± 11.46
21	2400	0.38	0.21 ± 0.1	44.36 ± 11.78
22	2500	0.45	0.21 ± 0.1	46.29 ± 12.04
23	2600	0.38	0.22 ± 0.09	48.11 ± 12.27

In petroleum-rich areas such as ASPA, increased ²²²Rn emission from drilling sites may facilitate the release of TENORM into the environment^51,52^. The residual accumulation of ²²²Rn and its progeny in groundwater, soil, and air may present enduring health threats to nearby communities, particularly in regions where residential areas are situated near petroleum operations. Consequences for risk management, the identified radiological characteristics indicate an urgent requirement for: Occupational safety: ²²²Rn surveillance, ventilation systems, and personal protective equipment (PPE) at drilling and production sites. Environmental monitoring: Regular assessments of ^222^Rn radioactivity concentrations in groundwater and soil gas to evaluate the dispersion beyond the petroleum field. Regulatory frameworks: Developing site-specific safety thresholds and mitigation techniques for ASPA, in light of the persistently high AED levels above suggested global limits^4,35,37,41,65^. The amalgamation of heightened α-index values, WL levels nearing occupational thresholds, and AED values substantially exceeding international guidelines strongly indicates that ASPA presents considerable radiological health risks. Inadequate management may elevate the risks of lung cancer and other ²²²Rn-related illnesses for petroleum workers and adjacent communities.

Combination variance of principal components PC1 (X-axis): Dominated by ²²²Rn concentration, surface exhalation rate, working level, and annual effective dose (all highly correlated in your earlier analysis). PC2 (Y-axis): Primarily influenced by mass exhalation rate and ^226^Ra concentration. Clustering data points spread along PC1 show increasing radiation levels (from left to right). Variables pointing in similar directions (e.g., ²²²Rn concentration, annual effective dose) are strongly positively correlated. Mass exhalation rate and ^226^Ra concentration form a separate cluster, consistent with their unique correlation pattern. Radiation exposure group: PC1 represents a composite index of ²²²Rn-related radiation exposure. Material properties group: PC2 captures ^226^Ra content and mass exhalation dynamics. Dimensionality reduction with 95%+ variance explained by PC1 + PC2 as shown in Fig. 8.

## Conclusions

The measured radioactivity concentrations of ^222^Rn and ^226^Ra across varying depths (100–2600 m) indicate a strong correlation between lithological variations and elemental composition. Lithology defined by mineralogical content, grain size, porosity, and permeability directly governs the emanation and migration of ²²²Rn from host rocks into pore spaces and fractures. Shale rich units (Dabaa, shale streaks in Abu Roash), higher radioactivity. Mixed sandstone shale units (Bahariya, Kharita), moderate radioactivity, increasing with clay content. Clean limestones units (Apollonia, Khoman), low radioactivity. Deeper formations may have slightly higher ^238^U enrichment in reducing environments (especially organic-rich shales), but lithology is the primary control. The data underscores the influence of lithology and geological formations on ²²²Rn and ^226^Ra distribution patterns. Deeper shale-sandstone sequences (e.g., Bahariya, Abu Roash) exhibit the highest ²²²Rn and ^226^Ra radioactivity concentrations, driven by their ^238^U content and structural porosity. These findings highlight the need for site-specific radiation safety protocols in hydrocarbon-rich and oil-sedimentary basins. Further studies integrating mineralogy and radioecology could refine risk assessments. Equations contextualized within IAEA and ICRP protocols were conducted directly to radiological impacts and environmental radioactivity. The descriptive statics Cross-validation methods ensure data reliability, variables, and units explicitly defined. The study provides a replicable framework for assessing NORM and TENORM risks in exploration and petroleum regions. Future studies should extend beyond the present dataset by incorporating advanced geochemical and isotopic analyses (AAS, ICP-MS, NAA, etc.) to better constrain heavy metal and radionuclide distributions. Integrating mineralogical characterization with hydrogeological and soil gas surveys will help clarify ²²²Rn migration pathways. Moreover, coupling field measurements with risk modeling will support the development of effective mitigation strategies for petroleum exploration environments. Comparative assessments across other petroleum provinces in Egypt are also recommended to establish a broader baseline for TENORM-related hazards doses.

## Supplementary Information

Below is the link to the electronic supplementary material.

Supplementary Material 1

## Data Availability

All results for the present study are included in this published version of article. The analyzed data is free available and would be requested on reasonable correspondence.
