# Supplementary material for: Nuclear track autoradiography for radon-related radiobiological hazards in Abu-Sannan Petroleum Area, Egypt
Source: Sci Rep. 2025 Nov 12;15:39573. doi: 10.1038/s41598-025-23659-8 (PMC12612125; doi:10.1038/s41598-025-23659-8)
Supplement: Supplementary file 1 — Supplementary Material 1 [file 41598_2025_23659_MOESM1_ESM.docx]

**Nuclear Track Autoradiography for Radon-Related Radiobiological Risks in Abu-Sannan Petroleum Area, Egypt**

**Calibration and Accuracy Control**

To ensure the accuracy of the measurements, calibration data for the CR-39 detector were considered as shown in figure 1. The detector's calibration factor (K), which relates track density to radon concentration, was determined elswhere previous experimental studies [1].


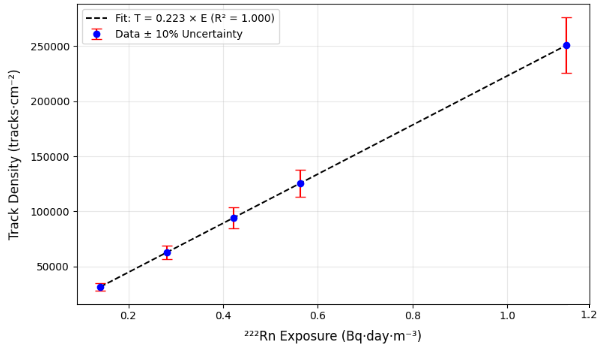


Fig. 1: Calibration curve of ^222^Rn for the CR-39 detector

The accuracy of the measurements, calibration data for the CR-39 detector were considered. The detector's calibration factor (K), which relates track density to ²²²Rn concentration, was determined elswhere previous experimental studies [2]. The minimal detectable limit (MDL) and measurement uncertainty were assessed to determine the dependability of the data. The sensitivity of the CR-39 NTD to ²²²Rn was determined to be 0.223 tracks/cm² per Bq.m⁻³. The Minimum Detectable Limit (MDL) and Measurement Uncertainty for the CR-39 detector were computed using the supplied calibration data. The MDL is the minimal ²²²Rn exposure that can be accurately differentiated from the lowest measured level, as determined by a calculation:

$MDL=\frac{3 \sigma_{blank}}{k}$ (10)

wher the standard deviation of blank readings (track density without ²²²Rn exposure) is denoted as k, which represents the calibration factor of CR-39 NTD.

The overall uncertainty in ²²²Rn exposure (E) is obtained from the uncertainty in track density measurement (σT=10.97%⋅T) and the uncertainty of the calibration factor, which is determined by weighted regression variance using error propagation for E=T/k:

$\frac{\sigma E}{E}=\sqrt{\left(\frac{\sigma T}{T}\right)^{2}+\left(\frac{\sigma k}{k}\right)^{2}}$ (11)

The minimum detectable limit for ²²²Rn concentration was determined to be 121.07 Bq.m^-^³, guaranteeing testing accuracy even at low ²²²Rn levels. The overall uncertainty in ²²²Rn measurements was 10.97%, consistent with IAEA norms [3-6].

References

1. Yousef, H.A., et al., *Assessment of radon in traditional building materials using polymeric nuclear track detector.* Radiation Effects and Defects in Solids, 2024: p. 1-10.

2. Shabaan, D.H., et al., *Radioanalysis of radon in dietary legumes using polymeric nuclear autoradiographic techniques.* Journal of Food Composition and Analysis, 2025. **147**: p. 108047.

3. IAEA, *Soil Sampling for Environmental Contaminants*. 2004, Vienna: International Atomic Energy Agency.

4. IAEA, *Analytical Methodology for the Determination of Radium Isotopes in Environmental Samples*. 2011, Vienna: International Atomic Energy Agency.

5. IAEA, *Nuclear Data for the Production of Therapeutic Radionuclides*. 2012, Vienna: International Atomic Energy Agency.

6. IAEA, *National and Regional Surveys of Radon Concentration in Dwellings*. 2014, Vienna: International Atomic Energy Agency.
